# DNA on the move: mechanisms, functions and applications of transposable elements

**DOI:** 10.1002/2211-5463.13743

**Published:** 2023-12-09

**Authors:** Michael Schmitz, Irma Querques

**Affiliations:** ^1^ Department of Biochemistry University of Zurich Switzerland; ^2^ Max Perutz Labs, Vienna Biocenter Campus (VBC) Austria; ^3^ Department of Structural and Computational Biology, Center for Molecular Biology University of Vienna Austria

**Keywords:** CAST, CRISPR‐associated transposons, CRISPR‐Cas systems, genome engineering, transposable elements

## Abstract

Transposons are mobile genetic elements that have invaded all domains of life by moving between and within their host genomes. Due to their mobility (or transposition), transposons facilitate horizontal gene transfer in bacteria and foster the evolution of new molecular functions in prokaryotes and eukaryotes. As transposition can lead to detrimental genomic rearrangements, organisms have evolved a multitude of molecular strategies to control transposons, including genome defense mechanisms provided by CRISPR‐Cas systems. Apart from their biological impacts on genomes, DNA transposons have been leveraged as efficient gene insertion vectors in basic research, transgenesis and gene therapy. However, the close to random insertion profile of transposon‐based tools limits their programmability and safety. Despite recent advances brought by the development of CRISPR‐associated genome editing nucleases, a strategy for efficient insertion of large, multi‐kilobase transgenes at user‐defined genomic sites is currently challenging. The discovery and experimental characterization of bacterial CRISPR‐associated transposons (CASTs) led to the attractive hypothesis that these systems could be repurposed as programmable, site‐specific gene integration technologies. Here, we provide a broad overview of the molecular mechanisms underpinning DNA transposition and of its biological and technological impact. The second focus of the article is to describe recent mechanistic and functional analyses of CAST transposition. Finally, current challenges and desired future advances of CAST‐based genome engineering applications are briefly discussed.

## Graphical abstract

Transposons are natural genome remodelers and gene insertion vectors. As such, they encode nucleic acid‐processing enzymes, whose molecular mechanisms and potential as genome engineering tools are yet only partially explored. CRISPR‐associated transposons hold great promise as molecular tools for RNA‐guided insertion of large transgenes. Here, we provide an overview of the mechanisms underpinning DNA transposition and of its technological impact.

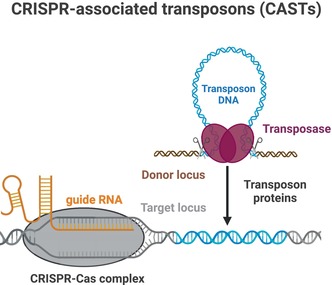

**Abbreviations**
attTn7: Tn7 attachment sites
bp: base pairs
Cas: CRISPR‐associated protein
CAST: CRISPR‐associated transposon
CRISPR: clustered regularly interspaced short palindromic repeats
crRNA: CRISPR RNA
DSBs: double‐strand breaks
*E. coli*
: *Escherichia coli*
HDR: homology directed repair
HGT: horizontal gene transfer
IHF: integration host factor
IR: inverted repeat
LE: left end
MGE: mobile genetic elements
NHEJ: non‐homologous end joining
ORF: open reading frame
PAM: protospacer adjacent motif
piRNA: PIWI‐interacting RNA
RE: right end
TE: transposable element
tracrRNA: trans‐activating CRISPR RNA
TS: target strand
TSD: target site duplication
uS15: bacterial ribosomal protein S15

Transposable elements (TEs) or transposons are prominent and ubiquitous residents of all genomes sequenced to date [1]. TEs are discrete DNA segments that can move from one location to another between and within their host genomes via a process termed transposition. Together with other mobile genetic elements (MGEs) such as plasmids and viruses, TEs constitute diverse mobilomes that generally occupy large fractions of the genomic DNA in prokaryotes [2] and eukaryotes [3].

## Transposition mechanisms

**Fig. 1:**
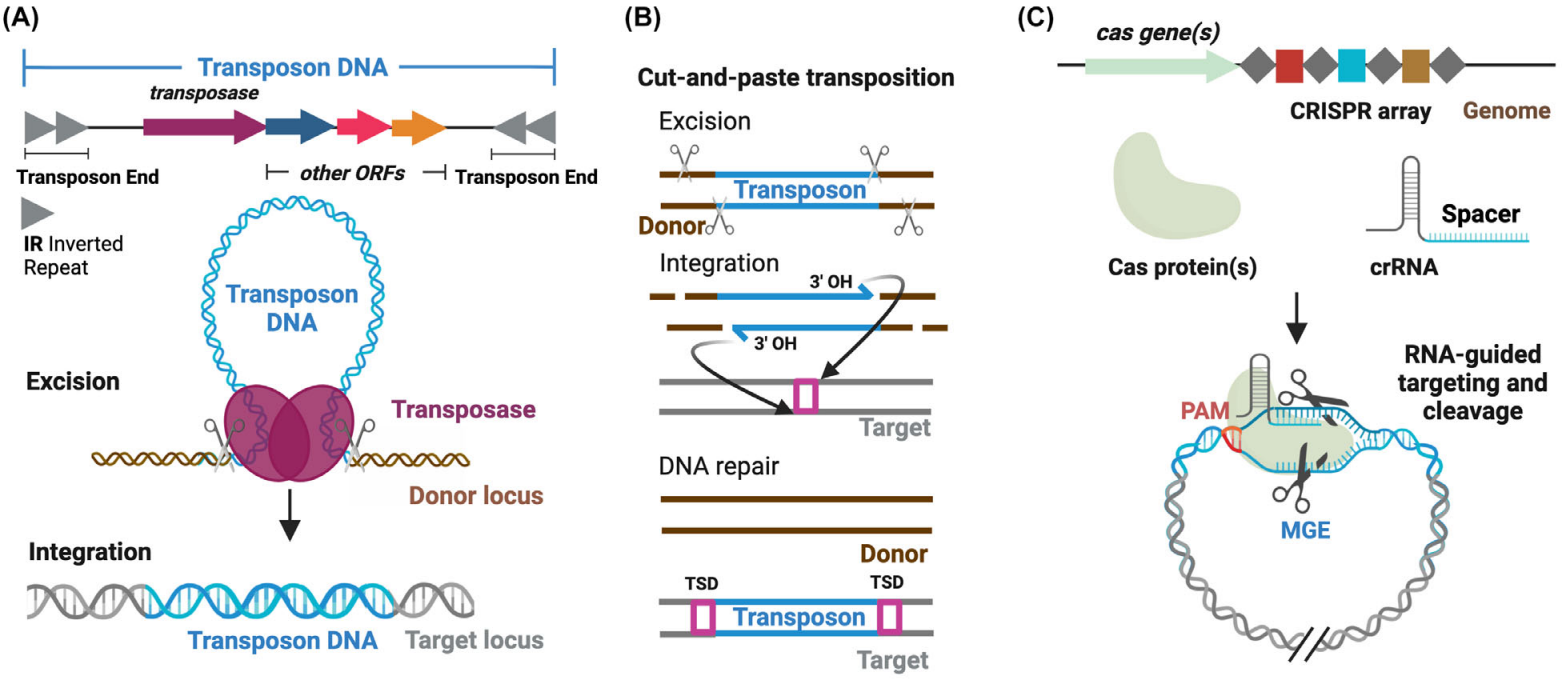
Mechanisms of canonical DNA transposons and CRISPR‐Cas systems. (A) Genetic architecture and simplified schematics of DNA transposons. (B) Mechanisms of cut‐and‐paste transposition. (C) Genetic architecture and simplified schematics of immunity provided by CRISPR‐Cas systems against mobile genetic elements (MGEs). crRNA, CRISPR RNA; ORFs, open reading frames; PAM, protospacer adjacent motif; TSD, target site duplication. Spacers are represented as squares, repeats as diamonds. Created with Biorender.com.

Based on their mechanism of movement, TEs can be grouped into two classes: Class I elements (or retrotransposons) that transpose via RNA intermediates, and Class II DNA transposons that rely solely on DNA molecules [4, 5]. This review focuses exclusively on Class II elements. Autonomous DNA transposons harbor at least one open reading frame (ORF) encoding a transposase enzyme that catalyzes the transposition reaction, flanked by two highly conserved transposon end sequences that encode binding sites for the transposase. Most DNA transposons feature characteristic inverted repeats (IRs) at the transposon ends (Fig. 1A). Non‐autonomous TEs lack an active transposase, but can be mobilized by enzymes supplied in *trans*, which is a key feature for the applicational usage of transposons [5], described later in this review.

The majority of DNA transposons move via a cut‐and‐paste pathway, whereby DNA double‐strand breaks (DSBs) are introduced at both transposon ends to physically liberate the transposon from its donor site (excision), followed by insertion of the transposon into a target locus (integration) (Fig. 1B) [6]. Excision requires nucleophilic reactions to break each DNA strand at both transposon ends; integration relies on transesterification resulting in the formation of new phosphodiester bonds between the transposon end and the target DNA strands without generating DSBs (Fig. 1B). Most cut‐and‐paste transposons generate target site duplications (TSDs) upon repair of staggered joins to the target DNA.

Some DNA transposition pathways require extensive DNA synthesis. In replicative transposition, for instance, only one strand at each transposon end is nicked and subsequently joined to the target DNA. Resulting co‐integrate intermediates can then be resolved by the host‐encoded DNA replication and repair machineries. Consequently, the transposon is not fully excised, but copied into the new target location (copy‐and‐paste transposition) [6].

## Biological impacts and regulation of transposons

The ability of transposons to mobilize their own DNA including additional cargo genes facilitates the spread of genetic information across the tree of life by horizontal gene transfer (HGT) mechanisms [7, 8]. By this, TEs are powerful evolutionary forces that can modulate the biology of their host organisms [9]. Among bacterial populations, for instance, the spread of antibiotic resistance and virulence genes encoded within transposons can have positive effects on the host survival [10]. However, transposon insertions can also disrupt the function of host genes or lead to harmful chromosomal rearrangements negatively impacting the host fitness, as demonstrated by more than 120 disease‐causing TE insertions in humans [11]. The integration of transposons into genomic ‘safe harbor’ sites is instead neutral to the host [12].

To protect their genomic integrity, organisms have evolved a broad set of mechanisms counteracting MGEs, including transposons. In animal species, this includes the PIWI‐interacting RNA (piRNA) pathway in which TEs are silenced co‐ or post‐transcriptionally by the RNA‐induced silencing complex guided by piRNAs [13]. In prokaryotes, clustered regularly interspaced short palindromic repeats (CRISPR)‐CRISPR‐associated protein (Cas) systems provide adaptive immunity against invading MGEs [14, 15]. In canonical CRISPR‐Cas systems, this is realized by insertion of invader‐derived nucleic acids fragments as spacers into a CRISPR array. Transcripts of the CRISPR array are processed into individual CRISPR RNAs (crRNAs) that guide Cas effector machineries to recognize complementary nucleic acid elements, ultimately targeting them for destruction (Fig. 1C) [16, 17]. Apart from host‐encoded regulatory mechanisms, some TEs also evolved strategies to fine‐tune their movement to preserve the host genome integrity and ultimately their own fitness. A prominent example is bacterial Tn7‐like elements [18]. These TEs tightly control their target site selection by transposon‐encoded protein factors (TnsD and TnsE) that mutually exclusively direct transposon integration into chromosomal safe harbor sites (Tn7 attachment sites – attTn7) [19, 20, 21] for transposon ‘homing’ (insertion into the host's bacterial chromosome) or other MGEs [22, 23, 24, 25], respectively.

Domestication of transposon‐derived proteins and nucleic acids fostered the evolution of new biological functions. Strikingly, adaptive immunity relying on CRISPR‐Cas systems in prokaryotes and the V(D)J recombination pathway in vertebrates, have arisen independently via domestication of recombinases encoded by unrelated TEs [26, 27, 28]. The evolutionary origin of CRISPR‐Cas systems from TEs support a ‘guns‐for‐hire’ model, in which the perennial arms race between bacteria and genetic invaders results in the shuffling of genes between offensive and defensive roles [28, 29].

## Genome engineering using TEs and CRISPR‐Cas systems

In addition to their biological roles as genome modifiers and gene transfer vehicles, TEs have been repurposed as efficient, broad‐host range gene insertion tools for genomic sequencing of bacterial communities [30], transgenesis of higher order organisms [31], and gene therapy in humans [32, 33]. In TE‐based applications, the transgene to be inserted is provided as a non‐autonomous transposon flanked by the transposon end sequences and mobilized by transposases conditionally supplied in *trans*. In present‐day gene therapy protocols, stable and efficient integration of therapeutic genes in the genome of human somatic cells is mostly achieved by means of lentiviral vectors [34]. Transposons constitute a valuable non‐viral alternative for gene integrations due to their high insertion efficiency, low immunogenicity, fairly unlimited cargo capacity (up to 20–150 kb long transgenes), and low complexity as well as costs for clinical implementation [35, 36, 37]. However, insertions by transposons or viral vectors occur at various sites and lack target selectivity, thus harboring a considerable risk of insertional mutagenesis [38] which limits their application for DNA integration at defined genomic sites. This is particularly concerning as regulated expression of therapeutic genes in specific genomic contexts is key to the success of gene therapy treatments [39].

Conversely, RNA‐guided CRISPR‐Cas effector nucleases, especially Cas9, have been leveraged to introduce DSBs at specific loci in the human genome with minimal requirements for the presence of a protospacer adjacent motif (PAM) (see Fig. 1C) [40, 41]. The introduction of genomic DSBs in mammalian cells stimulates endogenous non‐homologous end joining (NHEJ) or homology directed repair (HDR) pathways [42, 43]. As NHEJ mostly introduces insertions or deletions as a consequence of DSB repair, it is frequently used for gene knock‐outs. Triggering of HDR allows for recombination with a provided template DNA to introduce desired alterations to the targeted gene [44]. Due to the strict reliance on cell‐dependent DNA repair processes, introduction of genomic edits by RNA‐guided Cas9 and corrective DNA templates has limited efficiency for insertion of large transgenes (> 1 kb), especially in therapeutically relevant primary cells and post‐mitotic cells in which HDR is inefficient [45]. In general, introduction of genomic DSBs can lead to detrimental rearrangements as a consequence of both on‐target and off‐target chromosomal DNA breakage events [46, 47, 48]. Fusion strategies of nickase Cas9 to deaminases (base editing [49, 50]), reverse transcriptases (PRIME editing [42]), non‐LTR retrotransposon enzymes [51] or DNA polymerases (click editing [52, 53]) do not rely on the introduction of double‐stranded breaks any longer but are still limited in the size of the DNA sequences to be inserted. Hence, major limitations are still in place to fully revolutionize the field of genome engineering using TEs and CRISPR‐Cas systems. The initial discovery and experimental characterization of CRISPR‐associated transposons (CASTs) [26, 54, 55, 56] lead to the appealing hypothesis that these systems could overcome current challenges.

## CRISPR‐associated transposons: an unprecedented pathway of RNA‐guided DNA transposition

**Fig. 2:**
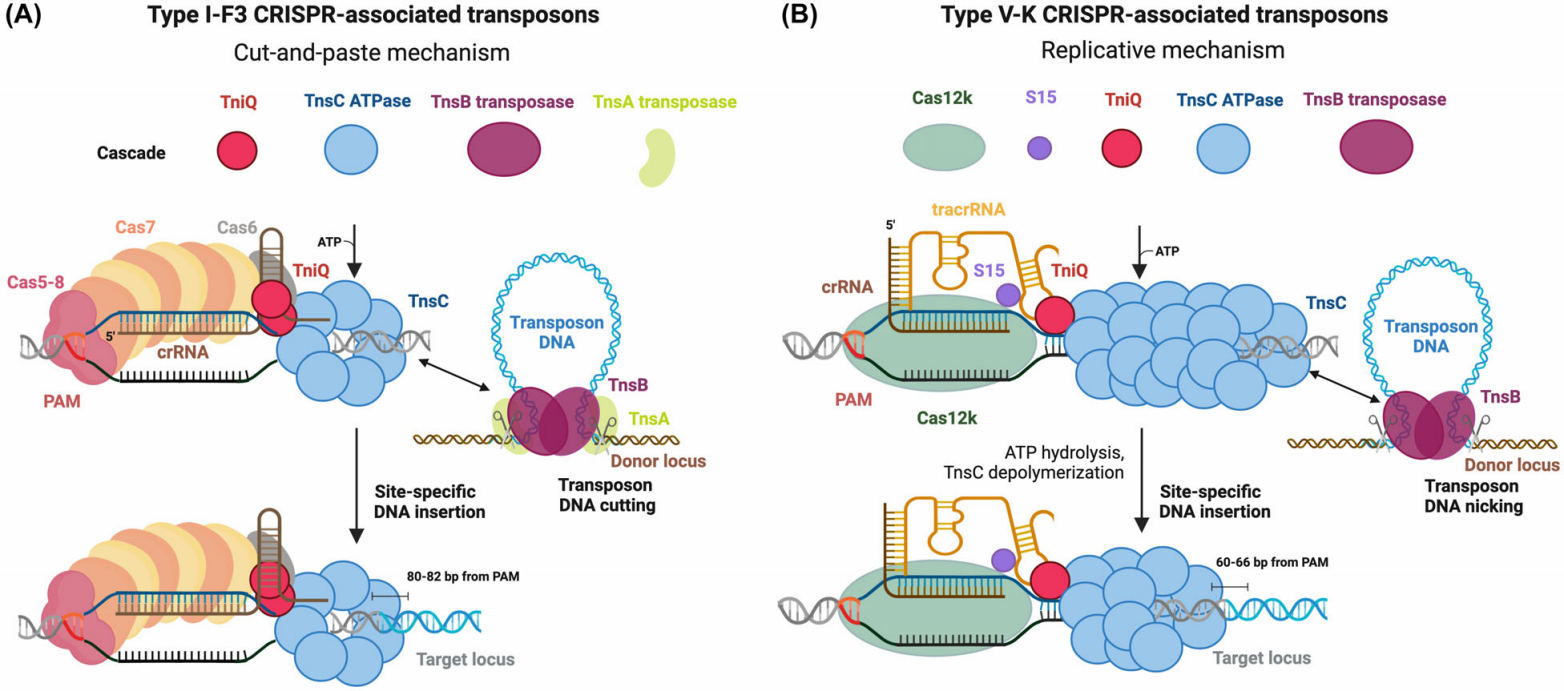
CRISPR‐associated transposons (CASTs). (A, B) Genetic architecture of type I‐F3 (A) and type V‐K (B) CASTs and proposed mechanisms of RNA‐guided transposition. PAM, protospacer adjacent motif. crRNA, CRISPR RNA. tracrRNA, tran‐sactivating CRISPR RNA. Created with Biorender.com.

Although canonical CRISPR‐Cas systems target MGEs for nucleolytic degradation (Fig. 1C), distinct Tn7‐like transposons have been discovered that coopted nuclease‐deficient CRISPR‐Cas systems to direct transposon DNA integration at a fixed distance from genetic loci specified by crRNA guides [26, 54, 55, 56, 57, 58, 59] (Fig. 2). Bacterial CRISPR‐associated transposons (CASTs) thus mobilize via a novel pathway of RNA‐guided DNA transposition and constitute the first natural systems capable of programmable, site‐specific DNA insertion. Thus far, type I (further subclassified into type I‐F [54], I‐B [59] and I‐D [57]) (Fig. 2A) and type V‐K [56] (Fig. 2B) CASTs have been experimentally characterized as RNA‐guided transposons. CASTs share a common genetic architecture, in which the left (LE) and right (RE) transposon ends flank genes encoding the transposition machinery – (*tnsA*)/*tnsB*, *tnsC*, *tniQ, (tnsD)* genes – and the CRISPR‐Cas system [26, 54, 55, 56, 57, 58, 59]. The CRISPR‐Cas modules are comprised by either a multisubunit Cascade complex in type I CASTs [54, 55, 57, 58, 59] (Fig. 2A) or a single Cas12k pseudonuclease effector that relies on an additional trans activating RNA (tracrRNA) molecule [26, 56] in type V‐K CAST systems (Fig. 2B). Target recognition by the CRISPR‐Cas effectors in a PAM‐dependent and RNA‐guided manner does not lead to the introduction of double‐stranded breaks in the target DNA but instead directs the transposition machinery to insert the transposon DNA in a small window at a fixed distance downstream of the selected target site [54, 56, 57, 59]. The core transposition machinery (the transpososome) in canonical Tn7‐like transposons and CAST systems is built of a varying number and type of transposase subunits [60, 61] (the DDE transposase TnsB and the type II restriction endonuclease‐like TnsA) and of TnsC ATPases [62]. Based on homology to the prototypical *Escherichia coli* Tn7 [63] and analysis of CASTs' excision [64] and integration events [65], TnsB catalyzes the 3′‐transposon DNA strand breakage and strand transfer reactions, whereas TnsA cleaves the 5′‐DNA strand of the transposon ends. Type I CASTs generally encode heteromeric (type I‐F3 [54, 55] and some type I‐B CASTs [59]) or naturally fused (type I‐D [57] and other type I‐B CASTs [59]) TnsA/TnsB transposases (Fig. 2A), whereas type V‐K CAST systems rely solely on the transposase TnsB [56] (Fig. 2B). The AAA^+^ ATPase TnsC regulates the activity of the transposases. TnsC binds the target DNA and is activated by interaction with a target selector. Notably, TnsC controls transposases by a yet unknown mechanism, such that the element is not excised before the target DNA is engaged, i.e. target recognition prompts excision.

The Tn7 transposon exclusively relies on protein factors for recognition of target sites, namely the target selectors TnsD and TnsE for the homing to the host genome [19, 20, 21] and targeting of other MGE [22, 23, 24, 25], respectively. In turn, all CAST systems experimentally investigated thus far utilize crRNA guides for targeting other MGEs. Homing of CAST systems to the host genome either occurs in an RNA‐guided manner (type I‐F3, I‐D and V‐K) [57, 59, 66] or is guided by TnsD‐like proteins (for a subset of type I‐B systems) [59]. crRNA guides used for homing of CASTs are either encoded as last spacers of the canonical CRISPR array or are stored in a minimal ectopic CRISPR array (Fig. 2B) and are sometimes mutated in their repeat or spacer segment [58, 59, 66]. CASTs often encode additional defense systems as cargo genes [67, 68] and are thus hypothesized to facilitate HGT of these systems within bacterial populations by hijacking other MGEs as shuttle vectors. Although initial insights into CASTs' regulation were reported [59, 66, 68], their exact function in prokaryotic biology is not fully understood at present.

## Molecular mechanisms of CAST transposition

Even though type I and type V‐K CASTs share a similar genetic architecture, their transposition mechanisms differ considerably. Mechanistic insights into type I CAST transposition have been mainly provided by structural and functional analyses of a type I‐F3 CAST derived from Vibrio cholerae (Tn6677) [54] (Fig. 2A) and a type I‐B element from *Peltigera membranacea*. The RNA‐guided DNA transposition relies on the concerted activities of the CRISPR‐Cas DNA targeting module, the Cascade, the zinc‐finger protein TniQ, the ATPase TnsC, and the transposases TnsA and TnsB [54]. Recognition of the PAM in the target DNA by the Cas8 subunit of the Cascade initiates the R‐loop formation that completes via potential intermediates [69, 70]. The type I‐F3 CAST TniQ forms a homodimer that is an integral component of the Cascade constituting the TniQ‐Cascade [69, 71, 72], while target DNA recognition and full R‐loop formation are required for recruitment of a TniQ monomer to the Cascade in type I‐B CAST [70]. Subsequent recruitment of further transposon components leads to transposon insertion circa 80–90 bp downstream of the PAM [54, 59]. Both type I‐F3 and type I‐B CAST TnsC have been shown to form discrete heptameric rings around the target DNA [70, 73]. The coordinated activity of TnsA/TnsB heterodimeric transposases in type I CASTs is thought to generate DSBs at both transposon ends, resulting in complete transposon excision via a cut‐and‐paste mechanisms and simple integration products at the target site (Fig. 1A) [18, 65]. Thus, type I CAST are ‘self‐sufficient’ and their transposition occurs with a high fidelity (~ 100%) [74]. Notably, host‐encoded Integration Host Factor (IHF) and ClpX proteins have been shown to enhance transposition of several type I‐F3 CASTs (IHF for prokaryotic cells [75], ClpX for prokaryotic and eukaryotic cells [76]), possibly by assisting transposon DNA bending and transpososome disassembly, respectively [75, 76].

Studies on a type V‐K CAST from the cyanobacterium *Scytonema hoffmanni* (Fig. 2B) revealed that the transposition of these elements requires the concerted activity of the pseudonuclease Cas12k, and the transposon proteins TniQ, TnsC and TnsB [56]. Cas12k‐mediated DNA targeting further relies on the tracrRNA [56] whose intricate fold serves as a scaffold to correctly position Cas12k and the crRNA guide for recognition of suitable DNA targets [77, 78]. Upon binding to a 5′‐GTN‐3′ PAM [56] in the target DNA, Cas12k initiates guide RNA hybridization with the matching target strand (TS) DNA, generating a partial R‐loop structure limited to 9 base pairs (bp) [77, 78]. Critical interactions with TniQ and TnsC elicit further guide RNA‐TS DNA hybridization and R‐loop completion to a 17 bp heteroduplex [79]. Apart from CAST‐encoded factors, the bacterial ribosomal protein S15 (uS15) was identified as a host factor that is part of the complex assembly and enhances the activity of the type V‐K CAST systems, establishing specific contacts with the tracrRNA and the crRNA‐TS heteroduplex [79]. A monomer of TniQ bridges between the CRISPR‐Cas machinery and the core transpososome by establishing physical interactions with TnsC [79] that in turn assembles into discrete ATP‐bound helical filaments remodeling the bound target DNA [77, 79, 80] and recruiting the TnsB transposase in close proximity to the selected target site [77, 81, 82]. Type V‐K CASTs lack the TnsA transposase [26] and are thus postulated to move via a replicative transposition pathway [18, 65, 83]. Concurrently, TnsB forms a tetramer when bound to the terminal transposon end sequences [82, 84] and interacts with the TnsC filament to trigger its disassembly by stimulating the ATPase activity of TnsC [77, 82], ultimately trimming the ATP‐bound TnsC filament to cover only the target DNA spanning to the integration site [81] 60–66 nt downstream of the PAM [56]. Notably, the level of on‐target insertions in type V‐K CAST systems is lower (~ 60%) [65, 74] compared to type I‐F systems as they promote a considerable amount of untargeted insertions as a result of a Cas12k‐ and crRNA‐independent transposition mechanism [65, 85, 86]. Further studies will be needed to address the molecular details underlying commonalities and differences in the currently described mechanistic models for type I and type V CASTs.

## Applications of CAST systems

The natural ability of RNA‐guided DNA integration by CAST systems could be repurposed for the insertion of large DNA payloads at specific genomic sites for genome editing, biotechnological and therapeutically relevant applications. At present, both type I and type V‐K CASTs have been harnessed in prokaryotes as site‐specific, homology‐independent and highly efficient DNA insertion tools to engineer bacterial strains in isolation or within communities [74, 87, 88, 89]. This allows a high degree of multiplexing [74, 87] and efficient in‐frame protein tagging [75], as well as to perform site‐ and species‐specific editing and thus monitoring of microbial populations [89].

The distinct requirements and mechanisms of natural CASTs have intrinsic advantages and disadvantages for their development as genome editing tools in heterologous hosts. While type V‐K CASTs are more compact and simpler due to their minimal CRISPR‐Cas effector (Cas12k), their host‐dependent replicative transposition mechanism results predominantly in undesired cointegration products [65, 83]. The addition of uS15 as a bona fide component [79] combined with the fusion of TnsB and a homing endonuclease, lead to a considerably improved fidelity (99.4%), to the conversion of the transposition mechanism to a cut‐and‐paste pathway, and established activity in mammalian cells, though at low efficiencies (~ 0.05%) [90]. Notably, the functionality of uS15 could not be replaced by its human counterpart [79].

In turn, uS15 does not serve as a functional component of type I CASTs as these elements do not involve a tracrRNA. Type I‐F3 CASTs have been shown to perform complete cut‐and‐paste transposition with their TnsA/TnsB transposases, resulting in simple insertion products with high fidelity (~ 100%) [65]. However, they require a multisubunit CRISPR‐Cas machinery to be delivered/expressed in heterologous hosts. Despite their complexity, type I‐F3 CASTs have been recently reported to be active in mammalian cells with efficiencies reaching ~ 5% [76]. The addition of host‐encoded factors (ClpX) was found to promote their activity in eukaryotic cells likely by supporting complex disassembly upon completed integration, which might also be of relevance for applications with other CAST systems [76].

As a methodological concept, future applications by CAST systems can in principle be developed for all scenarios that could benefit from precise insertion of large DNA payloads. Striking advantages of CAST‐based approaches in developing gene therapies, e.g. for monogenic disorders, are the one‐size fits all concept that no longer requires specific editing designs for individual mutations, but rather supplies a corrected version of the entire gene [91]. Moreover, advantages are the independence of HDR that further allows for genome engineering applications in primary and post‐mitotic cells.

Complementary approaches for the RNA guided insertion of large DNA fragments are further represented by initial fusion concepts of Cas9 to transposases [92, 93] or recombinases [94] and the more recently described PASTE [95] and twinPE [96] technologies. Both PASTE and twinPE technologies leverage the PRIME editor to first incorporate a suitable recombination site for an associated serine integrase that can subsequently insert a DNA template. Despite the multistep procedure and complex guide RNA design, efficiencies of up to 50–60% can be achieved in different mammalian cell lines [95, 96].

## Conclusions and outlook

Transposons are natural genome remodelers and gene insertion vectors that modify and transfer genetic information across the tree of life. As such, they encode a vast and diverse reservoir of nucleic acid‐targeting and ‐processing enzymes, whose molecular mechanisms and potential as genome engineering tools are yet only partially explored. Recently reported CRISPR‐associated transposons hold great promise as molecular tools for site‐specific insertion of large transgenes, but transplantation of these systems from the bacterial domain into higher (i.e. eukaryotic) organisms currently presents major challenges.

Future applications of CAST systems depend on robust reconstitution of RNA‐guided transposition in human cells. This would require the delivery of bacterial machineries to cell nuclei where targeted gene integration into chromatinized genomes needs to be carried out with high efficiency and fidelity. We envision that further engineering of type I and type V‐K systems can benefit from recent structural reports and the identification of prokaryotic host factors relevant for the activity in heterologous hosts, particularly in eukaryotic cells. Moreover, studies aiming at understanding and enhancing the activity of CASTs on chromatin will be pivotal for successful translational efforts. We expect that continued research on CASTs will expedite the development of highly specific, efficient and programmable gene insertion tools that could address unmet needs in genome editing, biotechnology and precision medicine.

## Conflict of interest

MS and IQ are named inventors on a patent application related to type V‐K CAST systems.

## Author contributions

MS and IQ jointly wrote the review.
